# Ssu72 Regulates Fungal Development, Aflatoxin Biosynthesis and Pathogenicity in *Aspergillus flavus*

**DOI:** 10.3390/toxins12110717

**Published:** 2020-11-13

**Authors:** Guang Yang, Xiaohong Cao, Ling Qin, Lijuan Yan, Rongsheng Hong, Jun Yuan, Shihua Wang

**Affiliations:** Key Laboratory of Pathogenic Fungi and Mycotoxins of Fujian Province, Key Laboratory of Biopesticide and Chemical Biology of Education Ministry, School of Life Sciences, Fujian Agriculture and Forestry University, Fuzhou 350002, China; guangyang@fafu.edu.cn (G.Y.); cxhcaoxiaohong@163.com (X.C.); lingqin@fafu.edu.cn (L.Q.); xiaoyan00998@163.com (L.Y.); michishimu5063@gmail.com (R.H.); yjmail2008@fafu.edu.cn (J.Y.)

**Keywords:** *A. flavus*, phosphatase, *ssu72*, pathogenicity, aflatoxins

## Abstract

The RNA polymerase II (Pol II) transcription process is coordinated by the reversible phosphorylation of its largest subunit-carboxy terminal domain (CTD). Ssu72 is identified as a CTD phosphatase with specificity for phosphorylation of Ser5 and Ser7 and plays critical roles in regulation of transcription cycle in eukaryotes. However, the biofunction of Ssu72 is still unknown in *Aspergillus flavus*, which is a plant pathogenic fungus and produces one of the most toxic mycotoxins-aflatoxin. Here, we identified a putative phosphatase Ssu72 and investigated the function of Ssu72 in *A. flavus*. Deletion of *ssu72* resulted in severe defects in vegetative growth, conidiation and sclerotia formation. Additionally, we found that phosphatase Ssu72 positively regulates aflatoxin production through regulating expression of aflatoxin biosynthesis cluster genes. Notably, seeds infection assays indicated that phosphatase Ssu72 is crucial for pathogenicity of *A. flavus*. Furthermore, the Δ*ssu72* mutant exhibited more sensitivity to osmotic and oxidative stresses. Taken together, our study suggests that the putative phosphatase Ssu72 is involved in fungal development, aflatoxin production and pathogenicity in *A. flavus*, and may provide a novel strategy to prevent the contamination of this pathogenic fungus.

## 1. Introduction

*Aspergillus flavus* is a saprophytic plant pathogenic fungus that infects a range of seed crops (maize, peanuts, cottonseed and tree nuts) before and after harvest [1,2]. In addition, *A. flavus* is also a human opportunistic pathogen, causing invasive aspergillosis in mammals and humans [3,4]. Aflatoxin (AF), which is mainly synthesised by *A. flavus*, is one of the most toxic and carcinogenic secondary metabolites in nature, posing a huge threat to economic development, food safety and human health [5,6]. Long-term intake of low doses of AFs may result in a number of health problems, such as growth impairment, lung and liver cancer or even death in many mammals and humans [7,8]. Therefore, it is essential to explain and clarify the regulatory mechanism of this fungus in pathogenicity and aflatoxin biosynthesis.

Previous studies have shown that aflatoxin biosynthesis and pathogenicity of *A. flavus* are regulated by multiple factors, such as temperature [9], water activity [10] and post-translational modifications (PTMs) including phosphorylation [11], acetylation [12], succinylation [13], methylation [14] and SUMOylation [15]. Reversible phosphorylation catalyzed by kinases and phosphatases, is one of the most common PTMs, and has been shown to regulate various biological processes [16,17]. In eukaryotes, reversible phosphorylation mainly occurs on three amino acids (serine, threonine and tyrosine) [18,19]. Phosphatase-mediated dephosphorylation plays a critical role in signal transduction in eukaryotic cells [20]. Based on sequence homology, structure and catalytic specificity, phosphatases can be classified into two major superfamilies: serine/threonine (S/T) phosphatases and tyrosine phosphatases (PTPs) [21]. The PTPs have demonstrated to be involved in the regulation of various cellular processes in eukaryotes, including cell cycle, signal transduction, transcriptional activation, development and secondary metabolism [22,23,24].

In eukaryotes, transcription of all coding genes is mainly regulated by RNA polymerase II (RNAP II) complex, which consist of 12 different subunits [25]. The carboxy-terminal domain (CTD) is one of the largest RNAP II subunits and composed of the evolutionarily conserved reiterate heptapeptide sequence (YSPTSPS), and reversible phosphorylation of this sequence is involved in distinct stages of transcription [26,27]. Ssu72 is identified as a highly conserved CTD phosphatase and negatively regulate the phosphorylation of specific Ser(5) and Ser(7) [28]. In *Saccharomyces cerevisiae*, Ssu72 is well studied due to its role in transcription initiation, mRNA processing, transcription termination and sister-chromatid cohesion [29,30]. In fission yeast, phosphatase Ssu72 is known to be essential for growth [31]. In addition, Ssu72 is found to be involved in RNA 3′ processing and the regulation of phosphate homeostasis in *Schizosaccharomyces pombe* [32,33]. In plant pathogenic fungus *Fusarium graminearum*, it has been demonstrated that the ortholog of phosphatase Ssu72 is involved in asexual development and virulence [34]. Despite the roles of phosphatase Ssu72 in yeast have been well studied, the function of Ssu72 in *Aspergillus* species is still poorly understood.

In this study, we identified a putative phosphatase Ssu72 of *A. flavus*, and then systematically characterized the function of Ssu72 in *A. flavus*. Our results reveal that the putative phosphatase Ssu72 plays critical roles in the regulation of fungal development, aflatoxin biosynthesis, stresses response and pathogenicity in *A. flavus*. This will provide a new insight that Ssu72 may be used as a potential target for preventing the hazard of *A. flavus*.

## 2. Results

### 2.1. Identification of Putative Phosphatase Ssu72 in A. flavus

**Figure 1:**
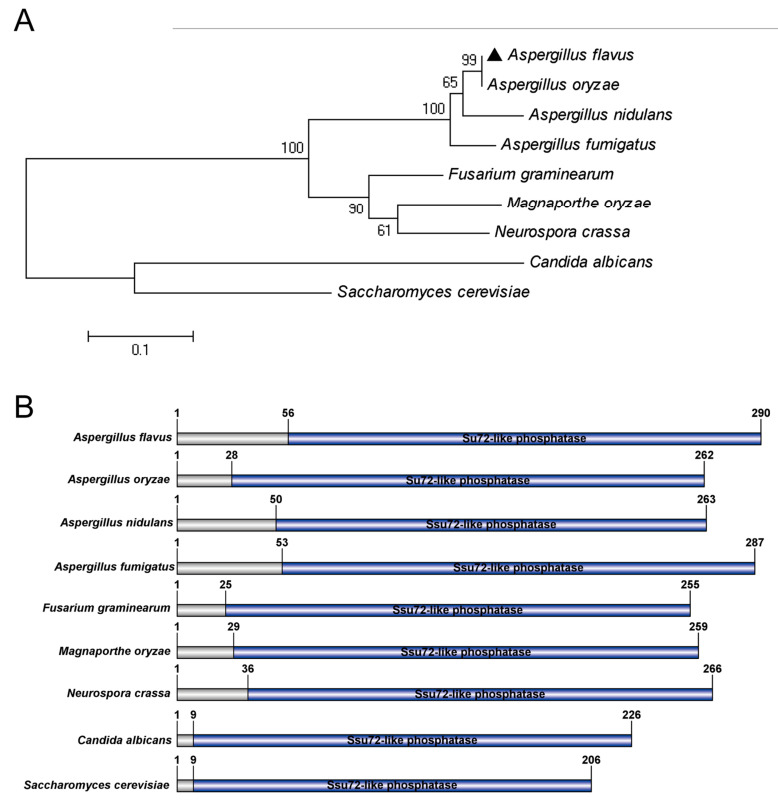
Identification of putative phosphatase Ssu72 in *A. flavus*. (**A**) Phylogenetic tree analysis of putative Ssu72 phosphatases from different organisms. The tree was generated by MEGA 5.1 software with Neighbour-joining and bootstrap method. (**B**) Domain structure analysis of putative Ssu72 phosphatase from different fungi. Protein structure was characterized by SMART and drew using DOG 1.0 software. The Ssu72-like phosphatase domain is shown in blue.

To identify the ortholog of Ssu72 phosphatase in *A. flavus*, the Ssu72 protein sequence of model organism *S. cerevisiae* was used to search with a basic local alignment search tool (BLAST) in the *A. flavus* genome database. A putative Ssu72 phosphatase protein in *A. flavus* was identified, predicting to encode 290 amino acids and present 45% homology with *S. cerevisiae* Ssu72. Then, a similar approach was performed to obtain Ssu72 homologous protein sequences in some fungi (*A. nidulans*, *A. fumigatus*, *F. graminearum*, *Magnapoethe oryzae*, *Neurospora crassa*, *Candida albicans*). Our sequence alignment results showed that the *A. flavus* Ssu72 protein displayed 99% similarity to *A. oryzae*, 95% similarity to *A. nidulans*, and 93% similarity to *A. fumigatus*. The phylogenetic analysis indicated that the Ssu72 protein is evolutionarily conserved in *Aspergillus* species (Figure 1A), and the *A. flavus* Ssu72 protein exhibited a high similarity with the ortholog of Ssu72 in *Aspergillus* species. Domain analysis revealed that the Ssu72 homologous proteins all contained a Ssu72-like phosphatase domain (Figure 1B), and this domain was mainly concentrated on the C-terminal of Ssu72 protein, suggesting that Ssu72-like phosphatase domain is highly conserved in fungi. These results indicate that the putative Ssu72 phosphatase protein is highly conserved in evolution and may have similar function in fungi.

### 2.2. Construction of the ssu72 Mutant Strains

**Figure 2:**
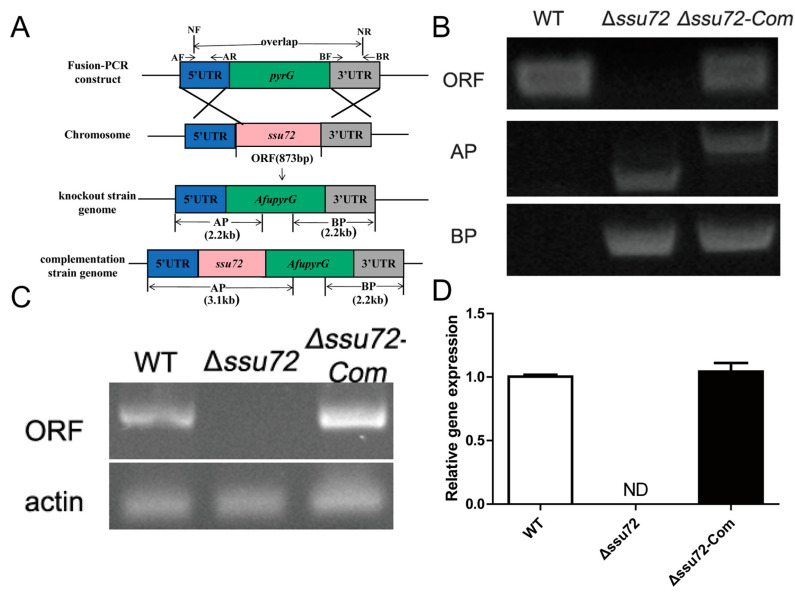
Construction and verification of *ssu72* mutants. (**A**) Strategy for deletion and complementation of phosphatase *ssu72* gene by using homologous recombination. (**B**) PCR analysis of deletion and complementation strains. ORF: open reading frame; AP: 5′UTR+*pyrG*; BP: 5′UTR+*pyrG*. (**C**) RT-PCR analysis was used to detect the expression levels of *ssu72* in different strains. *Actin* was used as reference gene. (**D**) qRT-PCR was performed to detect the transcript levels of *ssu72* in different strains. ND means not detected.

To investigate the potential function of Ssu72 phosphatase gene in *A. flavus*, the *ssu72* deletion (Δ*ssu72*) and the *ssu72* complementation (Δ*ssu72-Com*) strains were constructed by a homologous recombination approach (Figure 2A). The specific primers and sequence lengths are displayed in Figure 2A. For knockout strain construction, three fragments (1392 bp *ssu72* 5′UTR, 1220 bp 3′UTR and 1890 bp *pyrG*) were amplified with the specific primers and then fused together. *PyrG* from *A. fumigatus* was used to replace the *ssu72* gene, and open reading frame (ORF) of *ssu72* gene was reinserted into the genome of the knockout strain to construct complementary strains. Then, the deletion and complementation transformants were verified by polymerase chain reaction (PCR) analysis (Figure 2B), and the result showed that ORF fragment could be detected in WT (wild type) and Δ*ssu72-Com* strains, but not in Δ*ssu72* strain. While AP (5′UTR+*pyrG*) and BP (5′UTR+*pyrG*) fragments (Figure 2A) were amplified from *ssu72* deletion and complementation mutants but not from WT strain. Furthermore, expression levels of *ssu72* gene in WT, Δ*ssu72* and Δ*ssu72-Com* strains were analyzed by reverse transcription-PCR (RT-PCR) and quantitative real-time PCR (qRT-PCR), and the results indicated that the transcripts of *ssu72* were not detected in Δ*ssu72* strain, whereas *ssu72* gene was expressed in the WT and Δ*ssu72-Com* strains (Figure 2C,D). All these results showed that both *ssu72* knockout mutant (Δ*ssu72*) and *ssu72* complementation strain (Δ*ssu72-Com*) were successfully constructed.

### 2.3. Ssu72 Is Involved in Vegetative Growth in A. flavus

**Figure 3:**
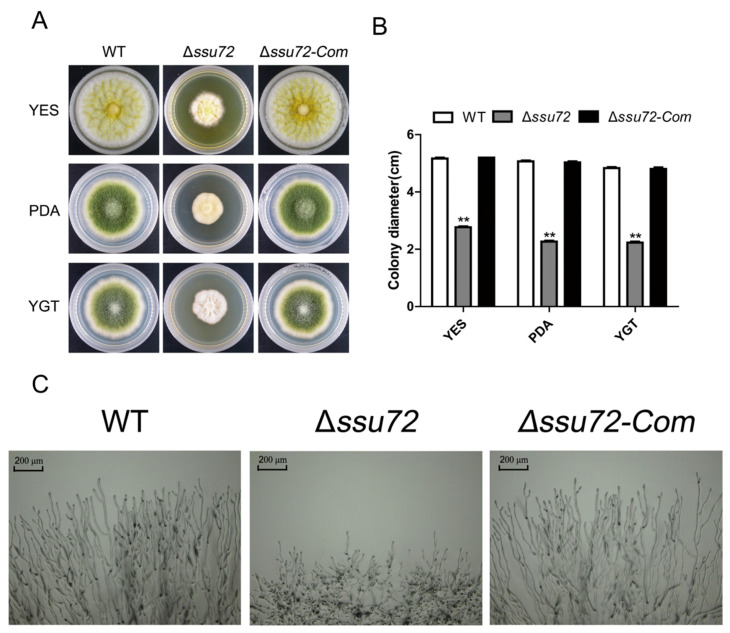
The function of *ssu72* in vegetative growth in *A. flavus*. (**A**) The colony phenotype of WT, Δ*ssu72* and Δ*ssu72-Com* strains grown on YES, PDA and YGT media for 5 days. All strains were cultured at 37 °C. (**B**) Colony diameter of all strains was measured on different media. Significant difference was analyzed by *t* test. ** *p* < 0.01 stands for significant difference. (**C**) Microscopic examination of mycelial tips in WT, Δ*ssu72* and Δ*ssu72-Com* strains, scale bars = 200 μm. Each experiment was repeated at least three times. Standard deviation is indicated by error bars.

To investigate the roles of *ssu72* in vegetative growth of *A. flavus*, the WT, Δ*ssu72* and Δ*ssu72-Com* strains were inoculated in yeast extract-sucrose (YES), potato dextrose agar (PDA) and yeast glucose trace-elements (YGT) media and cultured for 5 days. As shown in Figure 3A,B, our result revealed that the Δ*ssu72* mutant displayed significant growth reduction on YES, PDA and YGT media when compared with WT and complementation strains (Δ*ssu72-Com*). Moreover, microscopic examination showed that abnormal and shorter aerial hyphal were observed in Δ*ssu72* mutant, while the abnormal hyphal defect of Δ*ssu72* mutant was restored in the Δ*ssu72-Com* strain (Figure 3C). These observations suggest that phosphatase Ssu72 plays important roles in vegetative growth of *A. flavus*.

### 2.4. Ssu72 Is Critical for Conidiation in A. flavus

**Figure 4:**
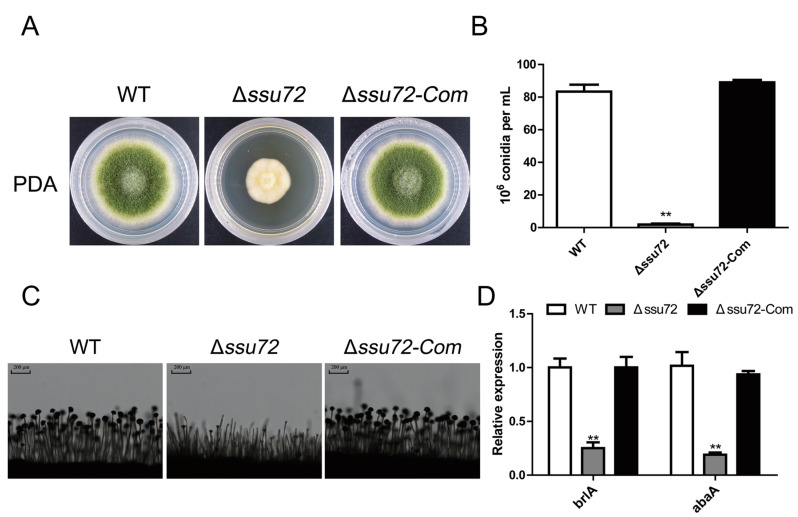
Defects of conidiation in Δ*ssu72* mutant. (**A**) Morphology analysis of WT, Δ*ssu72* and Δ*ssu72-Com* strains grown on PDA medium at 37 °C for 5 days. (**B**) Statistical analysis of the amount of conidia produced on PDA medium. (**C**) Conidiophores of all strains were observed by light microscope, scale bars = 200 μm. (**D**) Transcript levels of conidia key genes (*brlA* and *abaA*) in different strains after cultured for 48 h. *Actin* was used as reference gene. (** *p* < 0.01 means significant difference by *t* test.) Each experiment was repeated at least three times. Standard deviation is indicated by error bars.

Conidia is mainly produced by aerial hyphae and plays critical roles in asexual development and infection processes of *A. flavus* [35]. To assess the effect of *ssu72* in conidiation, the WT, Δ*ssu72* and Δ*ssu72-Com* strains were cultured on PDA medium for 5 days, and we found that the amount of conidia was obviously reduced in the Δ*ssu72* mutant in comparison with WT and Δ*ssu72-Com* strains (Figure 4A). About one tenth of conidia was observed in the knockout strain, when compared with the WT and complementation strains (Figure 4B). Furthermore, microscopic examination revealed that abnormal head of conidiophores was observed in Δ*ssu72* mutant (Figure 4C). To further explore the role of *ssu72* in conidiation, the expression levels of two key transcript factors (*brlA* and *abaA*) were analyzed by qRT-PCR. The consequence showed that the expression levels of *brlA* and *abaA* were both significantly down-regulated in Δ*ssu72* mutant when compared to WT and Δ*ssu72-Com* strains (Figure 2D). These results suggest that phosphatase Ssu72 is involved in the conidia production of *A. flavus*.

### 2.5. Ssu72 Is Required for Sclerotia Formation in A. flavus

**Figure 5:**
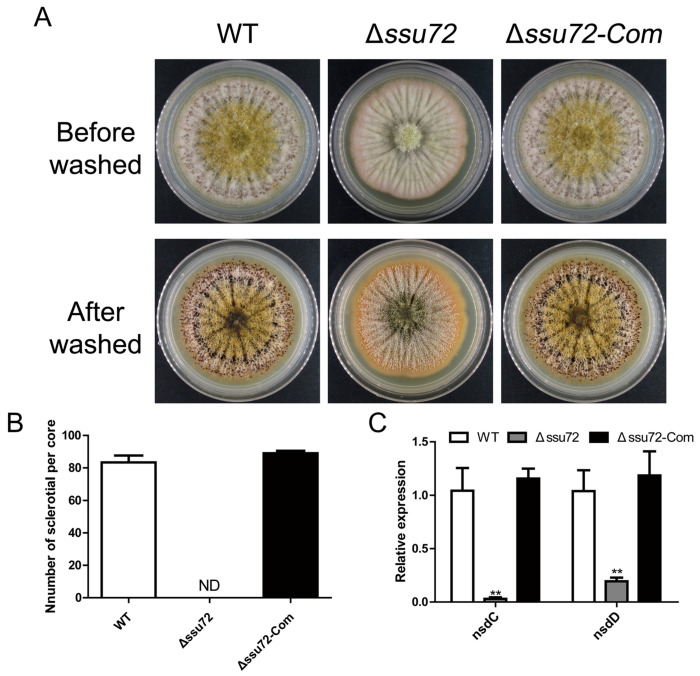
Phosphatase Ssu72 is essential for sclerotia formation. (**A**) Sclerotia formation of WT and *ssu72* mutants on WKM medium after 7 days. (**B**) Amount of sclerotia produced in different strains on WKM medium. (**C**) Expression levels of sclerotia formation key genes (*nsdC* and *nsdD*) in WT, Δ*ssu72* and Δ*ssu72-Com* strains cultured for 48 h. *Actin* was used as reference gene. (** *p* ≤ 0.01 means significant difference by *t* test.) ND means not detected. Each experiment was repeated at least three times. Standard deviation is indicated by error bars.

In *A. flavus*, a sexual structure-sclerotia is formed to survive under harsh environment [5]. To investigate the bio-function of *ssu72* in sclerotia formation, the WT, Δ*ssu72* and Δ*ssu72-Com* strains were cultured on the Wickerham (WKM) medium in the dark for 7 days. As shown in Figure 5A,B, lots of sclerotia were discovered in WT and Δ*ssu72-Com* strains. However, no sclerotia was observed in Δ*ssu72* strain. To further confirm these findings, qRT-PCR analysis was performed to analyze the transcript levels of two sclerotia formation key genes (*nsdC* and *nsdD*). The result indicated that the transcript levels of *nsdC* and *nsdD* were both dramatically reduced in the Δ*ssu72* mutants when compared to WT and Δ*ssu72-Com* strains (Figure 5C). All the above findings suggest that phosphatase Ssu72 is required for sclerotia formation of *A. flavus*.

### 2.6. Ssu72 Plays a Positive Role in Regulation of Aflatoxin Biosynthesis

**Figure 6:**
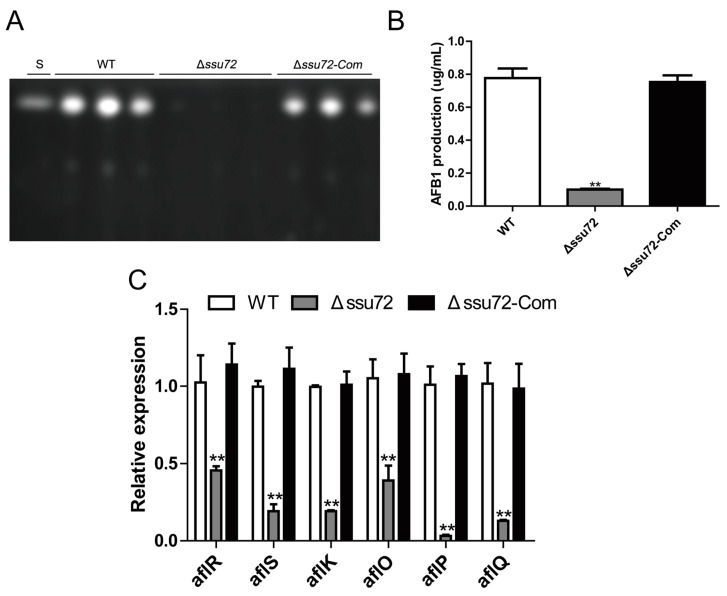
Analysis of aflatoxin production in the WT and Δ*ssu72* mutants. (**A**) Aflatoxins in the YES liquid medium were extracted in YES liquid medium for 6 days at 29 °C, and TLC was used to detect the aflatoxin production in each strain. S means AFB1 standard. The concentration of the AFB1 standard is 0.1 mg/mL. (**B**) Gene Tools software was used for quantification analysis of AFB1 as in (**A**). (**C**) Relative expression levels of aflatoxin biosynthesis cluster genes cultured for 48 h. *Actin* was used as reference gene. (** *p* ≤ 0.01). Each experiment was repeated at least three times. Standard deviation is indicated by error bars.

Aflatoxin (AF) is one of the most toxic secondary metabolites of *A. flavus*, and poses a huge threat to human health [36,37]. Thus, it is necessary to determine whether phosphatase Ssu72 is involved in aflatoxin biosynthesis. Then, the strains were cultured in YES liquid medium for 6 days, and thin-layer chromatography (TLC) analysis showed that deletion of *ssu72* gene resulted in a significant decrease in aflatoxin B1 (AFB1) production when compared to WT and Δ*ssu72-Com* strains (Figure 6A,B). Subsequently, qRT-PCR was performed to analyze the expression levels of AFB1 biosynthesis cluster genes. We found that the transcript levels of some key genes (regulatory genes *aflR* and *aflS*, structural genes *aflP*, *aflQ*, *aflO* and *aflK*) in Δ*ssu72* mutant were significantly lower than those in WT and Δ*ssu72-Com* strains (Figure 6C). Overall, these results indicate that phosphatase Ssu72 positively regulates AFB1 biosynthesis in *A. flavus*.

### 2.7. Ssu72 Contributes to Pathogenicity in Crop Seeds in A. flavus

**Figure 7:**
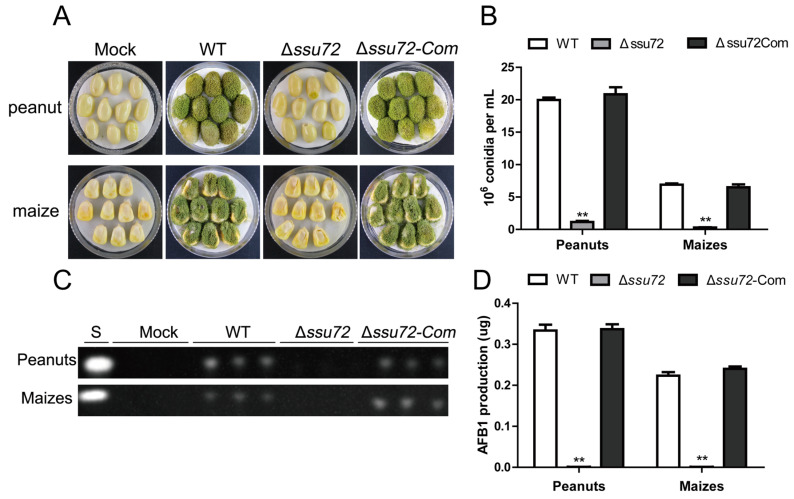
Analysis of seeds infection of WT, Δ*ssu72* and Δ*ssu72-Com* strains. (**A**) Sterile peanut and maize seeds were infected with WT, Δ*ssu72* and Δ*ssu72-Com* strains and cultured at 29 °C for 5 days. Mock means a blank control, peanuts and maize seeds were treated with the same amount of water instead of conidia. (**B**) Quantification analysis of conidia collected from the infected seeds. (**C**) TLC was used to detect the AFB1 production from the infected seeds. (**D**) Quantification of AFB1 as in (**C**). (** *p* ≤ 0.01). Each experiment was repeated at least three times. Standard deviation is indicated by error bars.

Since *A. flavus* infects crops and causes huge economic losses, the effect of phosphatase Ssu72 on the pathogenicity to crop seeds was characterized in this study. After cultivation for 5 days, a lot of conidia on surface of peanut and maize seeds were found in the WT and Δ*ssu72-Com* strain (Figure 7A). However, no obvious colonization was observed on peanuts and maize seeds after inoculated with Δ*ssu72* mutant (Figure 7A), indicating that deletion of *ssu72* resulted in a significant decrease in pathogenicity to peanut and maize seeds. Conidia were then harvested from the infected seeds, and Δ*ssu72* mutant produced less amount of conidia on infected seeds than WT and Δ*ssu72-Com* strain (Figure 7B). Subsequently, TLC assay was used to detect aflatoxin production in the infected seeds, and the result revealed that no AFB1 was observed in peanut and maize seeds infected with Δ*ssu72* strain (Figure 7C,D). All these data suggest that phosphatase Ssu72 contributes to its pathogenicity to crop seeds in *A. flavus*.

### 2.8. Ssu72 Response to Multiple Stresses in A. flavus

**Figure 8:**
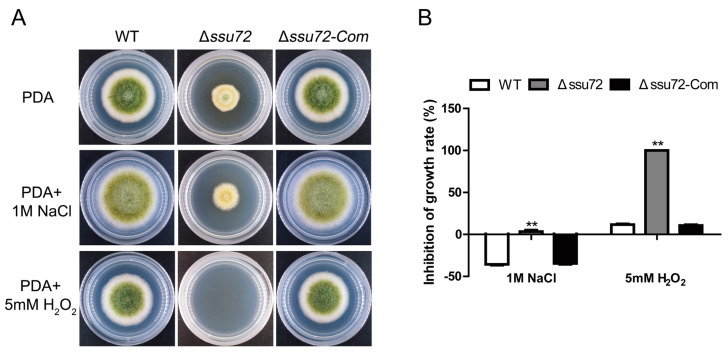
The Ssu72 is involved in osmotic and oxidative stresses response. (**A**) Colony morphology of WT, Δ*ssu72* and Δ*ssu72-Com* strains on PDA media added with 1 M NaCl or 5 mM H_2_O_2_ for 4 days. (**B**) Growth inhibition rate of all strains under osmotic and oxidative stresses. [inhibition of growth rate = (the diameter of untreated strain- the diameter of treated strain)/(the diameter of untreated strain) × 100%]. (** *p* ≤ 0.01 means significant difference by *t* test.) Each experiment was repeated at least three times. Standard deviation is indicated by error bars.

In *A. flavus*, phosphatases have been proven to be involved in multiple stresses response [38,39]. To explore the role of putative phosphatase Ssu72 in response to osmotic and oxidative stresses, the strains (WT, Δ*ssu72* and Δ*ssu72-Com*) were cultured on PDA medium supplemented with different stress reagents. As observed in Figure 8A,B, the relative inhibition of growth rate in Δ*ssu72* induced by 1 M NaCl was obviously higher than that of WT and Δ*ssu72-Com* strains, suggesting that Δ*ssu72* mutant displayed increased sensitivity to osmotic stresses. Additionally, the growth of Δ*ssu72* mutant was completely inhibited after added with higher osmotic stresses (5 mM H_2_O_2_) (Figure 8A,B). All these results demonstrate that Ssu72 participates in osmotic and oxidative stresses in *A. flavus*.

## 3. Discussion

As one of the largest RNAP II subunits, carboxy-terminal domain (CTD) is known to be involved in the regulation of transcriptional initiation/termination and pre-mRNA processing in eukaryotes [25,40]. Ssu72 is identified as a conserved CTD-related phosphatase and well characterized in yeast [33]. However, the biological functions of Ssu72 in other fungi, especially in *Aspergillus* species, are still unclear. In this study, a putative phosphatase Ssu72 of *A. flavus* was identified. The C-terminus of the ssu72 protein in fungi all contained a conserved Ssu72-like phosphatase domain, indicating that the ssu72 protein is evolutionarily conserved, and may have similar functions in fungi. In *S. cerevisiae*, the ssu72 protein has been shown to play important roles in regulation of multiple processes, so we speculate that Ssu72 may be involved in various processes in *A. flavus*. Next *ssu72* gene deletion and complementation strains were constructed to characterize its function in *A. flavus*. Our findings reveal that the putative phosphatase Ssu72 is involved in fungal development, aflatoxin production and pathogenicity of *A. flavus*.

Our previous studies have concluded that tyrosine phosphatases contribute to vegetative growth and conidiation in *A. flavus* [38]. In this study, our results demonstrated that knockout of putative phosphatase gene *ssu72* resulted in a severe defect in vegetative growth (Figure 3A), which is consistent with the results in budding yeast [31]. Our microscopic observation on hyphae tips showed that shorter aerial hyphal was found in Δ*ssu72* strain, which is in a good agreement with the above conclusion (Figure 3C). In addition, the amount of conidia was significantly reduced in Δ*ssu72* strain (Figure 4A), which is consistent with the defect that was observed in *ssu72* homolog mutants in *F. graminearum* and *N. crassa* [34,41]. This finding is well supported by the down-regulation of key transcription factors *brlA* and *abaA* [42]. Sclerotia is a sexual structure to survive in unfavorable environment in *A. flavus* [43]. However, no sclerotia was discovered in Δ*ssu72* strain when compared to WT and complementation strains (Figure 5A), suggesting that phosphatase Ssu72 is essential for sclerotia formation. Furthermore, transcript levels of *nsdC* and *nsdD* genes, which have been known to be critical for sclerotia formation [44], was also significantly decreased in Δ*ssu72* strain in this study. It is possible that deletion of *ssu72* decrease the expression levels of *nsdC* and *nsdD* genes, and then regulate the sclerotia production. Therefore, phosphatase Ssu72 plays important roles in the regulation of vegetative growth, conidiation and sclerotia formation in *A. flavus*.

As one of the most toxic secondary metabolites in nature, aflatoxin contamination poses a serious threat to food safety, human and animal health [45]. Recent studies have revealed that kinases and phosphatases play vital roles in regulation of aflatoxin production in *A. flavus* [39,46,47]. Here, we observed that aflatoxin production was dramatically reduced in Δ*ssu72* mutant, and this finding is in accordance with the down-regulation of aflatoxin biosynthesis regulatory and structural genes (Figure 6). A similar result was also found that deletion of *ssu72* homolog gene led to a lower production of mycotoxin-DON in plant pathogenic fungi *F. graminearum* [34]. A 70-kb DNA cluster is associated with aflatoxin biosynthesis, and regulatory and structural genes play important roles in this cluster [48]. As a conserved CTD phosphatase in fungi, Ssu72 has been proven to be involved in multiple cellular processes, including mRNA processing, transcription initiation and termination [29,32]. So we speculated that phosphatase Ssu72 may affect the transcription process of some key aflatoxin biosynthesis related genes, and then regulate the production of aflatoxin. These findings suggest that phosphatase Ssu72 positively regulates AFB1 biosynthesis in *A. flavus*.

Tyrosine phosphatases are well conserved and play critical roles in fungal pathogenicity in filamentous fungi [24,39]. In *F. graminearum*, homologues of Ssu72 have been demonstrated to be essential for plant infection [34]. However, the role of phosphatase Ssu72 in seeds infection is still unknown in *A. flavus*. Here, seeds infection assays showed that deletion of *ssu72* resulted in a severe defect in pathogenicity on maize and peanuts (Figure 7A), which is consistent with the previous finding in *F. graminearum* [34]. In addition, fewer conidia and aflatoxins were observed in the infected seeds of Δ*ssu72* mutant (Figure 7B,C). In *A. flavus*, pathogenicity is related to various factors, such as vegetative growth, conidiation, mycotoxins and environment stresses [43]. Therefore, we believed that the severe defects in development and aflatoxin production are the main reasons for the decreased pathogenicity to seeds. Taken together, these data suggest that phosphatase Ssu72 displays a vital role in pathogenicity to seeds of *A. flavus*.

Mitogen-activated protein kinase (MAPK) related phosphatases have been identified to be involved in multiple stresses response by regulating the phosphorylation level of MAP kinases (Hog1, Slt2 and Fus3) in *A. flavus* [39]. In this study, we found that the putative phosphatase Ssu72 is critical to respond to osmotic stress (Figure 8A). Similarly, the orthologue of Ssu72 has also been known to response to osmotic stress in *N. crassa*, and a higher phosphorylation level of Hog1 kinase was observed in *ssu72* deletion mutant [41]. Additionally, Δ*ssu72* mutants exhibited more sensitive to oxidative stress than WT and Δ*ssu72-Com* strains (Figure 8B). Reactive oxygen species (ROS) have been characterized to be highly related to oxidative stress [43]. In plant pathogenic fungi, elimination of ROS is critical to seeds infection [34]. Due to lower pathogenicity found in Δ*ssu72* strain, we speculate that ROS scavenging ability of Δ*ssu72* strain may be reduced. And this hypothesis is consistent with the result that the Δ*ssu72* mutant displayed more sensitive to oxidative stress in *A. flavus*.

In conclusion, a putative phosphatase Ssu72 was identified in *A. flavus*, and our results indicate that Ssu72 is involved in the regulation of development, aflatoxin biosynthesis and pathogenicity. This is the first report on the biofunction of phosphatase Ssu72 in *Aspergillus* species. We believe that our discoveries could improve the understanding of Ssu72 in filamentous fungi, and may provide a novel insight for developing new control strategies to this fungus.

## 4. Materials and Methods 

### 4.1. Strains and Culture Conditions 

Strains of *A. flavus* used and constructed were listed in Table 1. All strains were cultured on YES, PDA and YGT media for vegetative growth and conidiation analysis, and on WKM medium for sclerotia formation analysis [49]. YES liquid medium was used for aflatoxin production as described before [50].

### 4.2. Mutant Strains Construction

The homologous recombination approach was used to generate the *ssu72* knockout mutant (Δ*ssu72*) [4]. Three fragments (1392 bp *ssu72* 5′UTR, 1220 bp 3′UTR and 1890 bp *pyrG*) were amplified with specific primers and fused together. Then, the fusion PCR products were transformed into *A. flavus* CA14 protoplasts as described earlier [51]. As the *pyrG* gene is inserted into the knockout strain, the knockout strain could grow in a medium without urea. This feature could be used to screen the knockout strain. Afterwards, the *ssu72* complementation strain (Δ*ssu72-Com*) was constructed using a previously described method [50]. Briefly, an overlap PCR product which contains *ssu72* coding region was introduced into the Δ*ssu72* protoplasts. Finally, all the selected transformants were identified by PCR, and further confirmed by RT-PCR and qRT-PCR assays. At least two or three positive transformants were used for further phenotypic analysis. Primers are listed in Table 2.

### 4.3. Phenotpic Assays

To explore the roles of *ssu72* in growth and conidiation in *A. flavus*, 10^4^ spores were spotted on YES, PDA and YGT media, and cultured at 37 °C for 5 days. Colony diameter was measured after 5 days of cultivation. Subsequently, conidia were collected from PDA medium and quantified using a hemocytometer as previously described [52]. For sclerotia production assay, all strains were incubated on WKM medium at 37 °C for 7 days. Then, 75% ethanol was used to wash away the mycelia and conidia, and the sclerotia were harvested and counted by a light microscope as described earlier [53].

### 4.4. Aflatoxin Production Assays

To determine aflatoxin production, 10^4^ conidia of strains were inoculated on YES liquid medium and cultured at 29 °C for 6 days in the dark, then aflatoxins were extracted by chloroform according to a previously described approach [38]. TLC was performed to analyze aflatoxin in a solvent system (chloroform:acetone = 9:1) and examined under 365 nm UV light [13].

### 4.5. Pathogenicity Assays

Pathogenicity analysis on peanuts and maize seeds were conducted as described earlier [46]. Briefly, the sterilized crop seeds were inoculated with 10^6^ spores of each strain at 29 °C for 5 days. The infected seeds were collected and transferred to 50 mL centrifuge tubes with 15 mL sterile water. Then, the conidia amount and aflatoxin production of the infected seeds were analyzed as the methods mentioned before.

### 4.6. Stress Assay

To investigate the role of Ssu72 in various stresses response, the WT, Δ*ssu72* and Δ*ssu72-Com* strains were inoculated onto PDA medium supplemented with 1 M NaCl or 5 mM H_2_O_2_ at 37 °C for 4 days. The inhibition of growth rates was calculated as described before [38].

### 4.7. RNA Extraction and Quantitative Real-Time PCR Analysis

RNA extraction and cDNA synthesis were conducted according to the published references in our lab [39]. Mycelia were collected from PDA and WKM media cultured for 48 h, then TRIzol reagent (Biomarker Technologies, Beijing, China) was used for total RNA isolation. cDNA was synthesized with First-Strand cDNA Synthesis Kit (TransGen Biotech, Beijing, China). Subsequently, cDNA was used as a template for qRT-PCR analysis. All the qRT-PCR primers used in this study are listed in Table 3. The relative transcript levels of related genes were calculated with the 2^–ΔΔCt^ method [54], and *β-actin* was used as internal control.

## Figures and Tables

**Table 1:** Strains used in the study.

Strains	Genotype Description	Reference
*A. flavus* CA14 PTS	∆*ku70*, ∆*pyrG*	Our lab
wild-type (WT)	∆*ku70*, ∆*pyrG*::*AfpyrG*	This study
∆*ssu72*	∆*ku70*, ∆*pyrG*::*AfpyrG*, ∆*ssu72*	This study
∆*ssu72-Com*	∆*ku70*, ∆*ssu72*::*Aflssu72*, ∆*pyrG*::*AfpyrG*	This study

**Table 2:** Primers used in this study.

Primer Name	Sequence (5′–3′)	Application
*ssu72-*AF	AAACCGACCACGAAGACA	5 ‘UTR of *ssu72*
*ssu72-*AR	GGGTGAAGAGCATTGTTTGAGGCTCAAGGAGGCTGGAAGAT	
*ssu72-*BF	GCATCAGTGCCTCCTCTCAGACGGCTAGGGTCAACGAACA	3′UTR of *ssu72*
*ssu72-*BR	CCCTTCCCTCCTTCAGCA	
*pyrG-*F	GCCTCAAACAATGCTCTTCACCC	*fumigatus pyrG*
*pyrG-R*	GTCTGAGAGGAGGCACTGATGC	
*ssu72-*NF	GGTCCACTGGGTGGTAAT	Fusion PCR
*ssu72-*NR	GCACGATACAAGGCGATGG	
*ssu72-O*F	ACCCAGGAGCAACAGTCA	*ssu72* ORF verification
*ssu72-O*R	CCAGCCTTCAGAGTTATTCG	
P801-R	CAGGAGTTCTCGGGTTGTCG	Verification of AP and BP
P1020-F	CAGAGTATGCGGCAAGTCA	
*ssu72-Com-*AF	AACTAGTGAACACATCTTC	5′UTR of ∆*ssu72-Com*
*ssu72-Com-*AR	GGGTGAAGAGCATTGTTTGAGGCCCAATGTTCGTTGACCCTA	
*ssu72-Com-*BF	GCATCAGTGCCTCCTCTCAGACCTGTATGCAGATCCAAAT	3′UTR of ∆*ssu72-Com*
*ssu72-Com-*BR	CTTCTAAAGCTCCTATCC	
*ssu72-Com-*NF	TTCTGTTGGCCTGCGTAT	Fusion PCR
*ssu72-Com-*NR	GTATGCCTCTTGACTCCC	

**Table 3:** Primers used for qRT-PCR analysis.

Primer Name	Sequence (5′–3′)	Application
*ssu72-*F	GAGTCTTCAGACGGGACTGC	*ssu72* detection
*ssu72-*R	CACATTAGGTTGCGTGATGG	
*brlA*-F	GCCTCCAGCGTCAACCTTC	*brlA* qRT-PCR
*brlA*-R	TCTCTTCAAATGCTCTTGCCTC	
*abaA*-F	TCTTCGGTTGATGGATGATTTC	*abaA* qRT-PCR
*abaA*-R	CCGTTGGGAGGCTGGGT	
*nsdC*-F	GCCAGACTTGCCAATCAC	*nsdC* qRT-PCR
*nsdC*-R	CATCCACCTTGCCCTTTA	
*nsdD*-F	GGACTTGCGGGTCGTGCTA	*nsdD* qRT-PCR
*nsdD*-R	AGAACGCTGGGTCTGGTGC	
*aflR*-F	AAAGCACCCTGTCTTCCCTAAC	*aflR* qRT-PCR
*aflR*-R	GAAGAGGTGGGTCAGTGTTTGTAG	
*aflS*-F	CGAGTCGCTCAGGCGCTCAA	*aflS* qRT-PCR
*aflS*-R	GCTCAGACTGACCGCCGCTC	
*aflK*-F	GAGCGACAGGAGTAACCGTAAG	*aflK* qRT-PCR
*aflK*-R	CCGATTCCAGACACCATTAGCA	
*aflQ*-F	GTCGCATATGCCCCGGTCGG	*aflQ* qRT-PCR
*aflQ*-R	GGCAACCAGTCGGGTTCCGG	
*aflO*-F	GATTGGGATGTGGTCATGCGATT	*aflO* qRT-PCR
*aflO*-R	GCCTGGGTCCGAAGAATGC	
*aflP*-F	ACGAAGCCACTGGTAGAGGAGATG	*aflP* qRT-PCR
*aflP*-R	GTGAATGACGGCAGGCAGGT	
*actin*-F	ACGGTGTCGTCACAAACTGG	The endogenos gene
*actin*-R	CGGTTGGACTTAGGGTTGATAG	
